# Neuropathology in COVID-19 autopsies is defined by microglial activation and lesions of the white matter with emphasis in cerebellar and brain stem areas

**DOI:** 10.3389/fneur.2023.1229641

**Published:** 2023-07-13

**Authors:** Julian A. Stein, Manuel Kaes, Sigrun Smola, Walter J. Schulz-Schaeffer

**Affiliations:** ^1^Institute of Neuropathology, Medical Faculty of the Saarland University, Homburg, Germany; ^2^Institute of Virology, Saarland University Medical Center, Homburg, Germany; ^3^Helmholtz Institute for Pharmaceutical Research Saarland (HIPS), Helmholtz Centre for Infection Research, Saarbrücken, Germany

**Keywords:** COVID-19, neuropathology, macrophage, CD68, immunohistochemistry, CNS infection

## Abstract

**Introduction:**

This study aimed to investigate microglial and macrophage activation in 17 patients who died in the context of a COVID-19 infection in 2020 and 2021.

**Methods:**

Through immunohistochemical analysis, the lysosomal marker CD68 was used to detect diffuse parenchymal microglial activity, pronounced perivascular macrophage activation and macrophage clusters. COVID-19 patients were compared to control patients and grouped regarding clinical aspects. Detection of viral proteins was attempted in different regions through multiple commercially available antibodies.

**Results:**

Microglial and macrophage activation was most pronounced in the white matter with emphasis in brain stem and cerebellar areas. Analysis of lesion patterns yielded no correlation between disease severity and neuropathological changes. Occurrence of macrophage clusters could not be associated with a severe course of disease or preconditions but represent a more advanced stage of microglial and macrophage activation. Severe neuropathological changes in COVID-19 were comparable to severe Influenza. Hypoxic damage was not a confounder to the described neuropathology. The macrophage/microglia reaction was less pronounced in post COVID-19 patients, but detectable i.e. in the brain stem. Commercially available antibodies for detection of SARS-CoV-2 virus material in immunohistochemistry yielded no specific signal over controls.

**Conclusion:**

The presented microglial and macrophage activation might be an explanation for the long COVID syndrome.

## 1. Introduction

In January 2020, Zhu et al. (1) sequenced a novel coronavirus, the severe acute respiratory coronavirus 2 (SARS-CoV-2) as the pathogen of Coronavirus-19 disease (COVID-19) (1).

Although COVID-19 is largely considered a respiratory infection, neurological symptoms are fairly common: Pathognomonic early signs of infection are anosmia and ageusia (2, 3). Mild neurological manifestations include myalgia, headaches and dizziness (4). Severe neurological complications are mostly cerebrovascular events. However, disorders of the peripheral nervous system such as cases of Gullain-Barré-syndrome and facial neuropathy and inflammatory syndromes like encephalitis and encephalomyelitis have also been reported (5, 6).

The pathophysiology on how SARS-CoV-2 induces neurological symptoms is not well understood: Main competing hypotheses are neurotropism or effects mediated by the virus-induced cytokine storm (7–9).

Neuropathological findings in COVID-19 autopsies include microgliosis, astrogliosis, inflammatory infiltrates, hypoxic–ischemic lesions, edema and hemorrhagic lesions (10). The detection of viral proteins or nucleic acid in the central nervous system has been inconsistent and validity is controversially discussed (8, 10, 11).

This study aimed to investigate microglial and macrophage activation in 17 patients who died in the context of a COVID-19 infection in 2020 and 2021 through immunohistochemical analysis.

The lysosomal marker CD68 was used to detect the broad immune response to inflammatory processes in the brain. It is expressed by all cell lines of the mononuclear phagocyte system: meningeal macrophages, macrophages that migrated through bloodstream and activated phagocytizing microglia. Inflammatory glial and macrophage response was evaluated in distribution and intensity and compared to SARS-CoV-2-negative control patients.

Detection of viral proteins was attempted in different regions through multiple commercially available antibodies.

## 2. Materials and methods

### 2.1. Study design and participants

Patients (*n* = 17) who had died between April 2020 and June 2021 following a confirmed SARS-CoV-2 infection were autopsied at the Saarland University Medical Center (Homburg, Germany). Main inclusion criterion for this study was a confirmed diagnosis of SARS-CoV-2 infection via quantitative RT-PCR during lifetime. Clinical presentation was not considered for inclusion in this study. For comparison of inflammatory microglial and macrophage response, a control group of patients (*n* = 5) was autopsied that was not infected with SARS-CoV-2. Additionally, specimen of 12 control patients that died before 2020 was used as negative controls for the immunohistochemical detection of SARS-CoV-2 antigens. Health records of each patient were reviewed to gather information about comorbidities and hospitalization history. Basic characteristics of patients are summarized in Table 1. This study was approved by the local ethics committee of the Saarland Chamber of Physicians (approval number 21/23).

**Table 1:** Patient characteristics of COVID-19 patients (P) and control patients (C).

	Sex	Age (y)	Disease severity	Cause of death	Comorbidities	Neuropathological confounders
P 1	Male	87	NCU, oxygen therapy	Interstitial pneumonia with superinfection	Arterial hypertension	Cerebral amyloid angiopathy, B&B III
P 2	Male	55	ICU, ventilation (26 days), ECMO (9 days)	Interstitial pneumonia, sepsis with multiple organ failure	Diabetes mellitus type 2, arterial hypertension	Septic emboli in the cingulum bundle
P 3	Male	68	ICU, ventilation (20 days), sepsis	Interstitial pneumonia with superinfection	Arterial hypertension	Disseminated microembolisms, B&B I
P 4	Male	60	ICU, ventilation (41 days), ECMO (30 days), sepsis	Interstitial pneumonia with superinfection	Ex-smoker	Atrophy of the cerebellar vermis
P 5	Male	79	Hospitalization after CPR, ICU, ventilation (18 days), sepsis, kidney failure, organic brain syndrome	Interstitial pneumonia with superinfection, dilated cardiomyopathy	Arterial hypertension, coronary heart disease, diabetes mellitus, hyperlipidemia, severe arteriosclerosis	Multiple cavitary and cortical defects, hypoxic encephalopathy, severe gliosis
P 6	Female	96	PCR-confirmed infection, died 10 weeks post COVID, mild bronchitis 70 days before death	Pyelonephritis and urosepsis	Cardiac arrhythmias	Severe arteriosclerosis, Status cribrosus, perimortal global hypoxic–ischemic injury, B&B III
P 7	Male	69	ICU, ventilation (21 days), ECMO (16 days), pupils unresponsive to light before death	Interstitial pneumonia, dilated cardiomyopathy	Arterial hypertension, atrial fibrillation	None
P 8	Male	57	ICU, ventilation (1 day), ECMO (1 day)	Interstitial pneumonia, hypovolemic shock	Arterial hypertension, atrial fibrillation	Acute perimortal hypoxia
P 9	Female	69	Domestic quarantine, no symptoms, unsuccessful CPR	Hypovolemic shock after acute aortic dissection	Arterial hypertension, systemic vasculitis (glucocorticoid therapy)	Arteriosclerosis, acute perimortal hypoxia
P 10	Male	89	Domestic quarantine, mild bronchitis	Cardiac arrest following hypertrophic cardiomyopathy	Hypertrophic cardiomyopathy	Arteriosclerosis, ischemic strokes, CERAD B, B&B III
P 11	Male	84	Domestic quarantine, mild bronchitis	Interstitial pneumonia, sepsis	Severe arteriosclerosis	Cerebral amyloid angiopathy, CERAD B, B&B V
P 12	Female	74	Incidental positive test before autopsy, no hospitalization	Interstitial pneumonia, lung cancer with cachexia	Arterial hypertension, coronary heart disease, hyperlipidemia, diabetes mellitus, nicotine abuse, status post myocardial infarction and stroke, end stage lung cancer	Arteriosclerosis and status cribrosus, cortical microinfarctions, cerebral amyloid angiopathy, B&B III, cerebral metastasis frontoparietal, perimortal hypoxia
P 13	Male	72	ICU, ventilation (1 day), sepsis, unsuccessful CPR	Cardiac arrest following superinfected interstitial pneumonia	Bronchial carcinoma, diabetes mellitus	Suspected early stage of corticobasal degeneration (CBD)
P 14	Female	87	NCU, mild bronchitis	Infectious toxic cardiovascular failure	Heart failure, 2-vessel coronary heart disease, status post myocardial infarction, arterial hypertension	Cavitary defects, arteriosclerosis, CERAD 0, B&B III
P 15	Male	61	ICU, ventilation (66 days), ECMO (48 days), sepsis, acute kidney failure, CPR	interstitial pneumonia with bacterial superinfection, cardiovascular failure	Adiposity, spinal stenosis	Arteriosclerosis, B&B I
P 16	Male	81	NCU	Infectious toxic cardiovascular failure, interstitial pneumonia	Severe peripheral arterial occlusive disease, myocardial hypertrophy	Arteriosclerosis, cerebellar atrophy, cerebral amyloid angiopathy, CERAD B, B&B III, perimortal hypoxia
P 17	Female	87	NCU, died 4–5 weeks post COVID unrelated to COVID	Acute liver failure following hip surgery	Dementia, osteoporosis, trigeminal neuralgia	Arteriosclerosis and status cribrosus/lacunaris, perimortal hypoxia
C 1	Male	66	ICU, ventilation (38 days), sepsis, multiorgan failure	Severe superinfected interstitial pneumonia (Influenza)	Coronary heart disease, atrial fibrillation, cardiomyopathy, nicotine and alcohol abuse	Cerebellar atrophy
C 2	Male	70	NCU, deterioration of general condition	Severe pulmonary embolism	Arterial hypertension, 3-vessel coronary heart disease, polycystic kidney disease with kidney transplant, DLBCL	Arteriosclerosis with status cribrosus/lacunaris, multiple cavitary defects, microinfarctions
C 3	Male	66	NCU	Tumor cachexia	Metastasized prostate cancer	Multiple microinfarctions, arteriosclerosis and status lacunaris
C 4	Male	55	ICU, sepsis, CPR	Severe pulmonary embolism	Adiposity, diabetes mellitus, arterial hypertension	Arteriosclerosis and status cribrosus, multiple infarctions
C 5	Female	47	Preclinical CPR, ICU, ventilation (1 day), cerebral edema, multiorgan failure	Hypovolemic cardiovascular failure (hemorrhagic gastritis)	Alcohol abuse, anemia, adiposity	Cerebellar atrophy, perimortal global hypoxia, status cribrosus

y, years; NCU, normal care unit; ICU, intensive care unit; B&B, staging of tau pathology according to Braak et al. (12); ECMO, extracorporal membrane oxygenation; CPR, cardio-pulmonal reanimation; PCR, polymerase chain reaction; CERAD, amyloid plaque score according to The Consortium to Establish a Registry for Alzheimer’s Disease (CERAD) (13); DLBCL, diffuse large B-cell lymphoma.

### 2.2. Sampling and specimen processing

All brains and other samples were examined macroscopically and then fixed in buffered 4% formaldehyde for at least 14 days before cutting. Then formalin-fixed paraffin-embedded tissue (FFPE) blocks were taken. The sampling sites were gyrus frontalis medius, gyrus cinguli, gyrus parietalis inferior, area striata, anterior striate, basal ganglia, amygdala, thalamus, hippocampus, cerebellar vermis, cerebellar hemisphere, mesencephalon, pons, medulla oblongata and trigeminal ganglion.

### 2.3. Staining

Hematoxylin–eosin (HE) staining according to standard procedures and immunohistochemical staining were performed on 1–3-μm-thick FFPE tissue sections.

Sections were deparaffined and rehydrated. Heat antigen retrieval was performed by steaming at 98°C in target retrieval solution (TRS) pH 6.1 (Dako, Cat.-No. S1699) for 30 min or pH 9 (Dako, Cat.-No. S2367) for 15 min. Sections were then allowed to cool down. Peroxidases were blocked by incubation in 1% H_2_O_2_ for 10 min (20 min for CD68) at room temperature either prior (all antibodies except for CD68) or after heat antigen retrieval. Immunohistochemical stainings were performed using the coverplate system (Thermo Fisher Scientific) and a Dako staining kit. Sections were subsequently incubated for 45 min at room temperature with the primary antibody. The primary antibodies and specific pretreatments used for detecting microglia/macrophage activity and SARS-CoV-2 antigens are summarized in Table 2. For the neuropathological diagnosis and to determine confounders the following antibodies were used: beta-amyloid (Zytomed Systems, Cat.-No. Z932002-Y, 1:1.000), tau (ThermoFisher Scientific, Cat.-No. MN1020, 1:1.000), alpha-Synuclein antibody 10D2 (Roboscreen Diagnostics, Leipzig, Cat-No. 0102004703, 1:1.000), GFAP (Dako, Cat.-No. M0761, 1:50), MNF-116 (Dako, Cat.-No. M0821, 1:500), CK7 (Dako, Cat.-No. M7018, 1:1.500), CK20 (Dako, Cat.-No. M7019, 1:50), TTF-1 (Dako, Cat.-No. M3575, 1:100), CD3 (Dako, Cat.-No. M7254, 1:25).

**Table 2:** Primary antibodies and specific pretreatments.

Primary antibody	Species	Clone	Manufacturer	Dilution	Pretreatment	Target
CD68	Mouse monoclonal	PG-M1	Agilent/Dako, Santa Clara, CA, USA Cat.-No.: M0876	1:100	TRS pH 9,0	Macrophage-restricted form of the CD68 antigen
Abcam 3A2	Mouse monoclonal	3A2	Abcam, Cambridge, UK Cat.-No.: ab272420	1:100	TRS pH 6,1	Human coronavirus SARS spike glycoprotein
SB-NC	Mouse monoclonal	Clone #05	Sino Biological, Peking, China Cat.-No: 40143-MM05	1:200	TRS pH 6,1	SARS-CoV nucleocapsid Cross-reactivity: SARS-CoV-2 nucleocapsid protein
CoV-2-S1A9	Mouse monoclonal	1A9	GeneTex, Irvine, USA Cat.-No.: GTX 632604	1:250	TRS pH 6,1	SARS-CoV-2 spike protein (S2 subunit)
Novus-NC	Rabbit polyclonal	-	Novus Biologicals, Centennial, USA Cat.-No.: NB100-56576	1:250	TRS pH 6,1	SARS-CoV-2 nucleocapsid protein
SB-Spike	Rabbit polyclonal	-	Sino Biological, Beijing, China Cat.-No.: 40150-T62-CoV2	1:500	TRS pH 6,1	SARS-CoV spike glycoprotein

Cat.-No., catalog number; TRS, target retrieval solution.

All antibodies were diluted in Dako REAL antibody diluent (Dako, Cat.-No. S2022). Following three washes with wash buffer (Dako, Cat.-No. S3006), 3-amino-9-ethylcarbazol (AEC) was used for visualization of the antibody reaction in the case of CD68- and SARS-CoV-2-related primary antibodies. For all other immunohistochemical procedures the Dako REAL EnVision HRP kit (Cat.-No. K5007) was used according to the manufacturer’s instructions. Using both methods, brown staining was rated as a “positive signal.” Sections were then counterstained with Mayer’s haemalum (Sigma-Aldrich, Cat.-No. 1.09249), mounted and coverslipped.

### 2.4. Microscopic analysis

HE and immunohistochemistry sections were evaluated by one board-certified neuropathologist and one medical student with concurrence.

First, an orienting histomorphological assessment was carried out on the HE stained sections. Subsequently, CD68-immunoreactivity was assessed. Slides were screened at low magnification and areas with the most pronounced changes were used for quantification. Two distinct patterns were observed: the presence of diffuse parenchymal microglial activation and the presence of perivascular macrophage activation. A semiquantitative categorization, similar to the grading of Yang et al. (8), for activation was applied: 1 = “mild” was attributed to detectable microgliosis, which is atypical for healthy tissue; 2 = “moderate” was equal to a process typical of pathological changes; 3 = “severe” was a distinct pathological process such as clusters of microglia or macrophages. Like Yang et al. (8), the spatial context was applied to determine the CD68+ myeloid cell type.

To obtain a definite diagnosis and determine confounders, selected areas were stained to evaluate infarctions, lymphocytic infiltrates, and neurodegenerative changes. The β-amyloid pathology was scored according to the CERAD amyloid plaque score (13) and the tau pathology was scored according to Braak et al. (12).

For the evaluation of the specificity of SARS-CoV-2 antibody pattern of various antibodies (Table 2), the reaction pattern evident in COVID-19 cases were checked for appearance in control cases.

Images were acquired with an Olympus BX 40 microscope, equipped with a digital microscope camera using the Olympus cellSens Entry software.

### 2.5. Statistical analysis and graphical illustration

Both distinct patterns, diffuse parenchymal microglial activation and perivascular macrophage activation, were scored from 0 to 3 individually and subsequently aggregated as neuropathological lesion score (“NP lesion score”). Range of possible NP lesion score values is 0 to 6. Areas with the most pronounced changes compared to control patients were selected for graphical illustration. Groups were composed according to clinical variables. Ten areas were chosen for characterizing the lesion pattern in the brain of COVID-19 patients: the white matter of the medial frontal gyrus, the white matter of the anterior cingulate gyrus, the putamen at the level of the anterior striate, the amygdala, the white matter besides the hippocampus at the level of the lateral geniculate, the white matter of the cerebellar hemisphere, the cerebellar nuclei, the white matter of the pons, pontine nuclei and the pyramidal tract at the level of the midbrain. With the regions on the x-axis, line charts were plotted with the mean value and the range of grading scores for each analysis. For group composition, various clinical aspects were applied to determine influencing factors.

Statistical analysis and graphical illustration were performed using Microsoft Excel (version 16.57).

## 3. Results

### 3.1. COVID-19 patients show a distinct pattern

**Figure 1:**
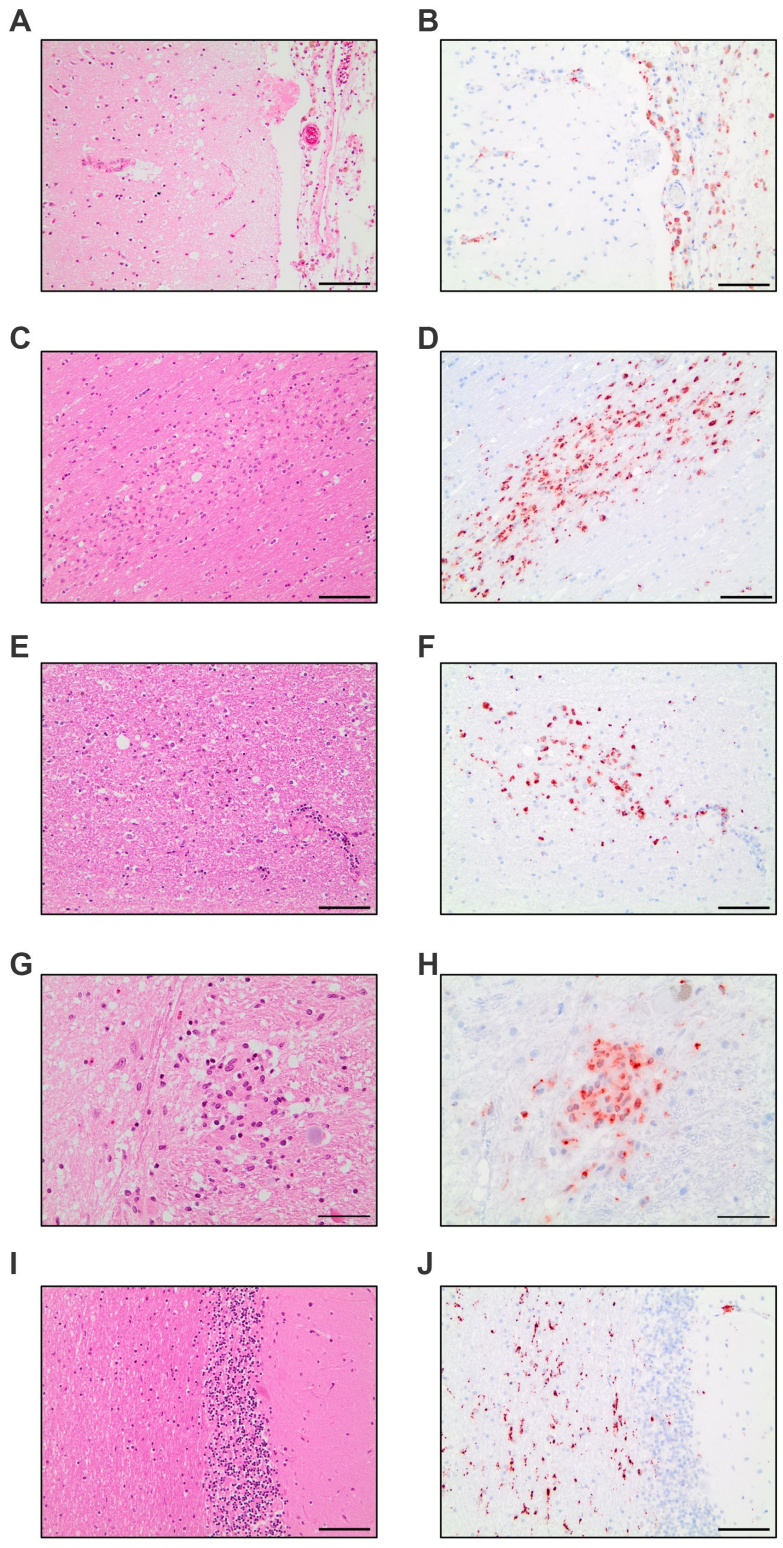
Compilation of CD68-immunohistochemistry findings in COVID-19 patients. Meningeal macrophage infiltration in HE-staining **(A)** and CD68-IHC **(B)**. Macrophage clusters in the white matter of the cingulate gyrus in HE-staining **(C)** and CD68-IHC **(D)**. Macrophage clusters in the white matter of the pons in HE-staining **(E)** and CD68-IHC **(F)**. Macrophage cluster in the nucleus of solitary tract in HE-staining **(G)** and CD68-IHC **(H)**. Diffuse parenchymal microglial activity in the white matter of the cerebellum in HE-staining **(I)** and CD68-IHC **(J)**. **A–F**,**I**,**J**: Scale bar = 100 microns, **G,H**: Scale bar = 50 microns.

The evaluation of all 17 COVID-19 patients versus control patients yielded three distinct pattern which were present throughout the COVID-19 patients: diffuse parenchymal microglial activity, pronounced perivascular macrophage activation and macrophage clusters (illustrated in Figure 1).

The diffuse microglial pattern was most pronounced in the cerebellar nuclei, followed by white matter areas of the cerebrum and brain stem areas surrounding pontine nuclei. The perivascular macrophage component showed the same distribution across analyzed regions of the brain but had less variation in intensity across patients. COVID-19 microglia/macrophage activation can therefore be considered a pathology of the white matter with emphasis in brain stem and cerebellar areas.

**Figure 2:**
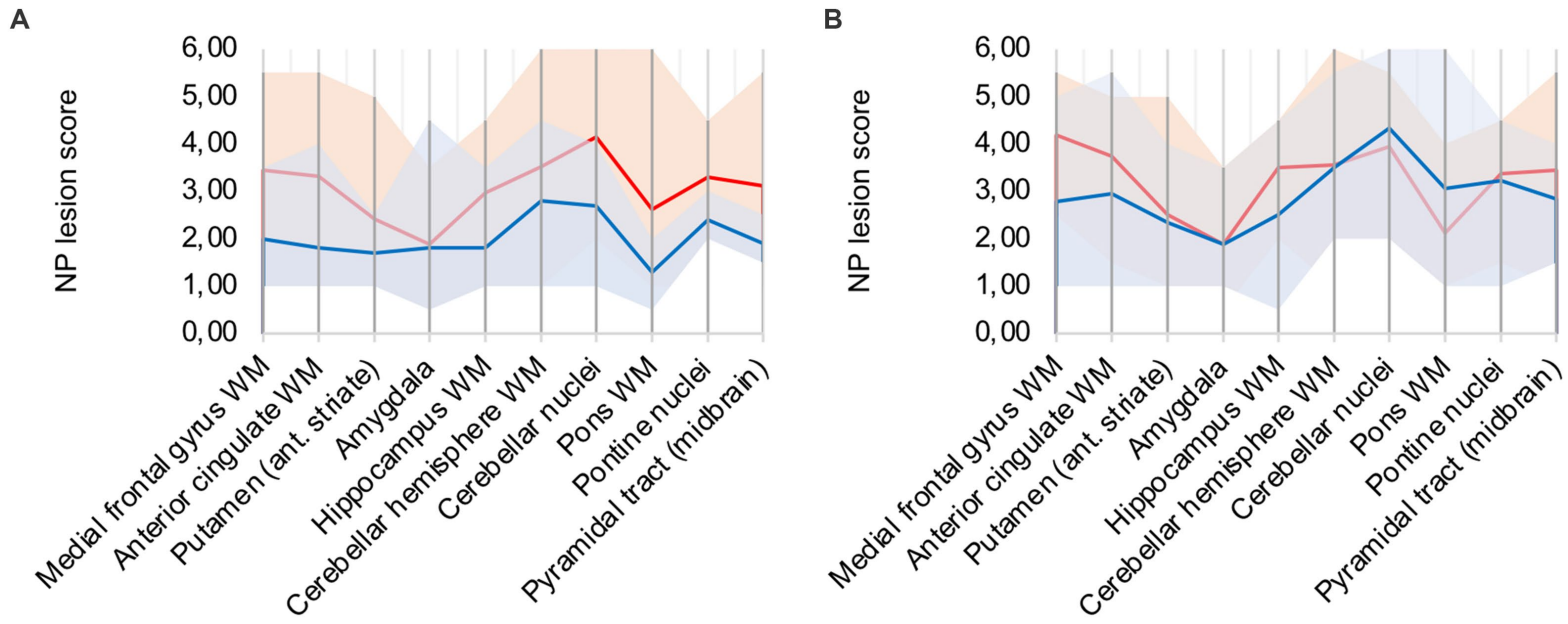
**(A)** Lesion pattern of COVID-19 patients (red) vs. control patients (blue), **(B)** COVID-19 patients with “severe course of disease” (red) vs. COVID-19 patients with “mild course of disease” (blue).

In comparison to control patients, the mean sum of both patterns showed a consistently elevated level of microglia/macrophage activation in all areas apart from the amygdala (illustrated in Figure 2A).

### 3.2. No obvious association between disease severity and neuropathological changes

COVID-19 patients were divided into two groups: “Severe course of disease” was defined as patients that required intensive care and ventilation at least for a short time (patients 2, 3, 4, 5, 7, 8, 13, and 15). These patients were critically ill according to the National Institutes of Health’s Covid-19 treatment guidelines (14). Remaining patients did not require hospitalization or were treated on a normal care unit and were grouped as “Mild course of disease.”

Analysis of lesion patterns yielded no obvious association between disease severity and neuropathological changes (illustrated in Figure 2B). While COVID-19 patients with “severe course of disease” showed higher microglial/macrophage activity in white matter areas of the cerebrum, patients with “mild course of disease” showed higher activity in the cerebellum and brain stem areas.

### 3.3. Patients with macrophage clusters show advanced stage of inflammatory microglial activity

CD68-positive clusters were detected in 10 patients (patients 2, 3, 4, 7, 8, 10, 11, 13, 14, 15), representing 59% of analyzed COVID-19 autopsies. Clusters in the white matter were most frequent (*n* = 8), most often in white matter areas of the cerebrum (*n* = 6) followed by the white matter of the brain stem (*n* = 5), the internal capsule/pyramidal tract (*n* = 3) and the cerebellar white matter (*n* = 1). Clusters were also frequently observed in the nuclei of the brain stem (*n* = 4), the basal ganglia (*n* = 1) and in the cerebellar nuclei (*n* = 1). Occurrence of clusters could not be associated with a severe course of disease.

**Figure 3:**
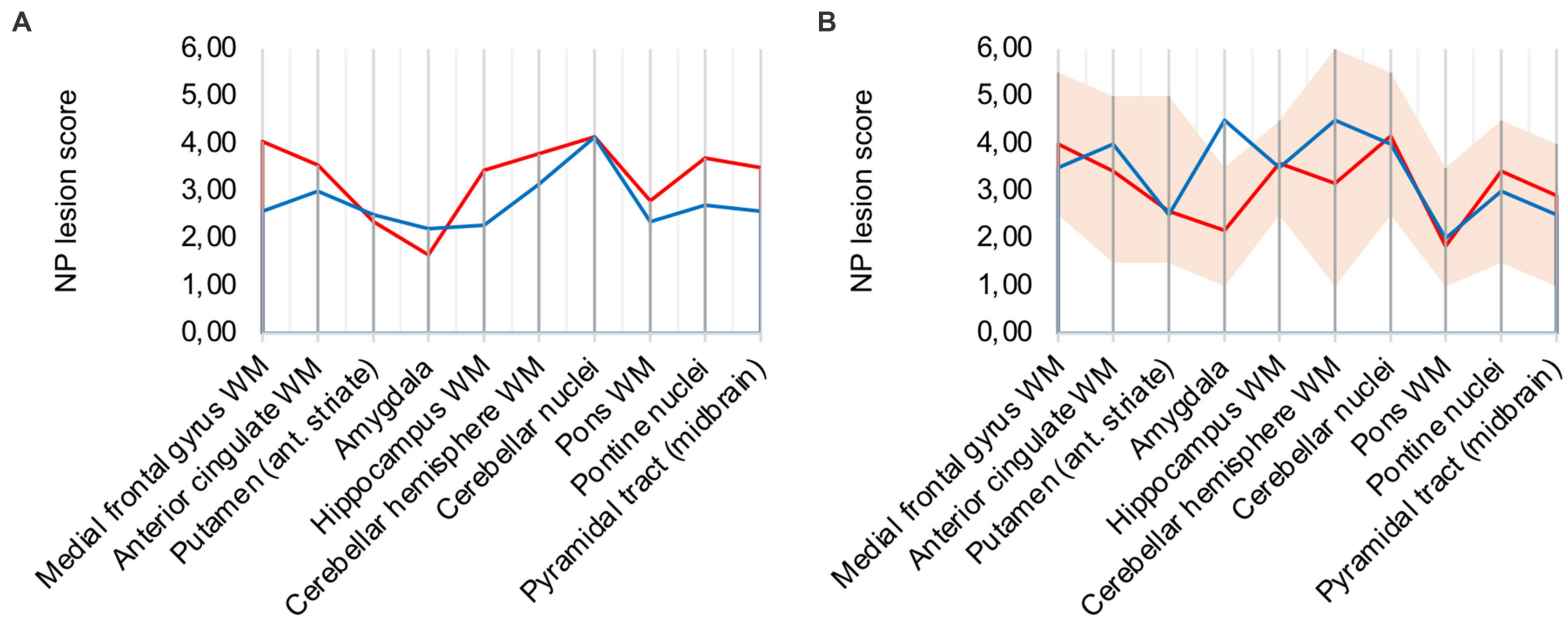
**(A)** Lesion pattern of COVID-19 patients with CD68-positive clusters (red) vs. without CD68-positive clusters (blue), **(B)** Effects of long-term ventilation on lesion pattern – COVID-19 patients (red) vs. the long-term ventilated Influenza patient (blue).

Analysis of all 10 COVID-19 patients with clusters against the remaining cohort shows that regions with frequent occurrence of clusters had higher microglia/macrophage activation in the mean (illustrated in Figure 3A). It can therefore be assumed that clusters represent a more advanced stage of microglial and macrophage activation.

### 3.4. COVID-19 neuropathology shows similarities to severe influenza

To evaluate intensive-care treatment and long-term ventilation as confounders to the described COVID-19 neuropathology, we compared severe Influenza (control patient 1), who was ventilated for 38 days, to six COVID-19 patients (patients 2, 3, 4, 5, 7, 15) that were ventilated for an average period of 32 days. Like most of the selected COVID-19 patients, control patient 1 suffered a septic shock.

Analysis of lesion patterns suggests that neuropathological changes in severe COVID-19 are comparable to severe Influenza (illustrated in Figure 3B). In both cases microglia/macrophage activation in white matter areas of the cerebrum and cerebellum was elevated. Notably, control case 1 showed pronounced microglial and macrophage activation in the amygdala. It also presented macrophage clusters in the brain stem, however not in white matter areas of the cerebrum.

### 3.5. Hypoxic damage is different from COVID-19 neuropathology

**Figure 4:**
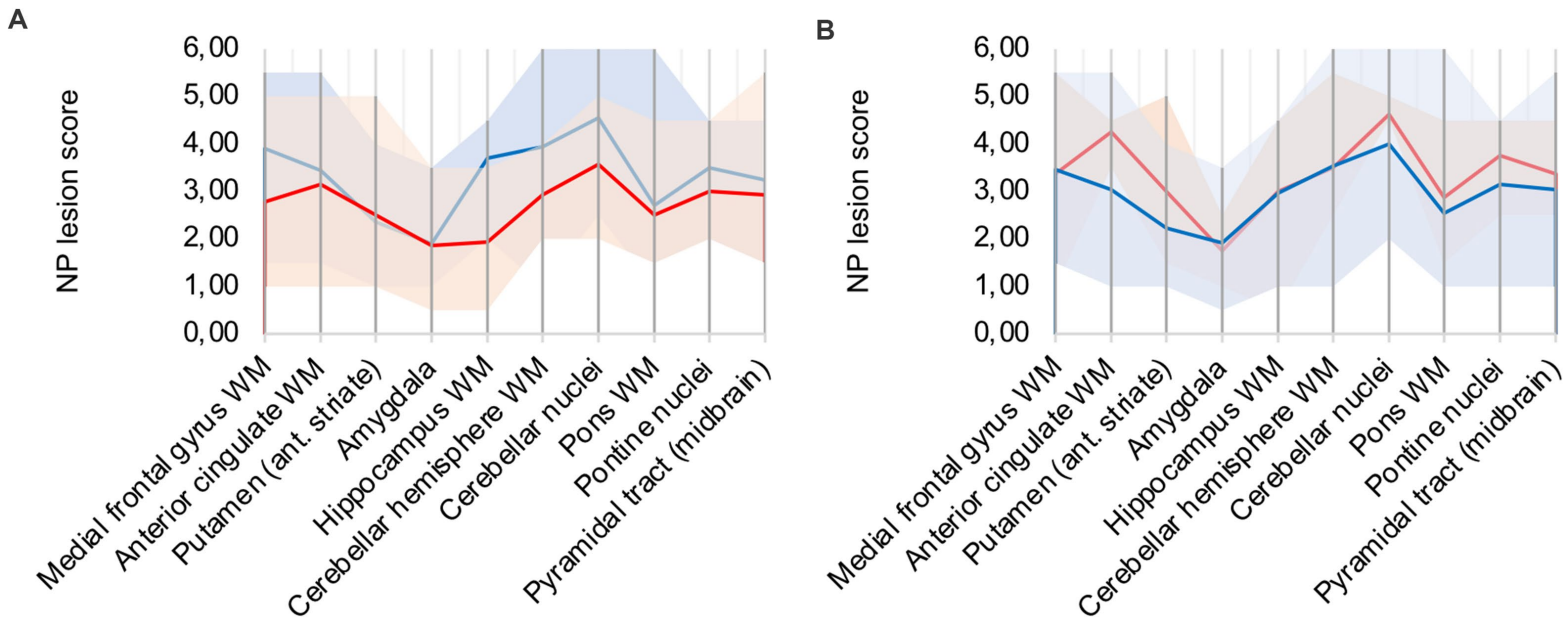
**(A)** Hypoxic (red) vs. non-hypoxic (blue) COVID-19 cases, **(B)** Influence of cardio-pulmonal reanimation – COVID-19 patients (red) vs. control patients (blue).

A frequent co-pathology (*n* = 7; ≈ 41%) of analyzed COVID-19 patients was hypoxic damage, frequently diagnosed through neuronal cell death in the CA1 region of the hippocampus. Hypoxic COVID-19 patients (patients 5, 6, 8, 9, 12, 16, 17) were compared to the rest of the cohort (illustrated in Figure 4A): microglial and macrophage activity was less pronounced in hypoxic patients, notably hippocampus, cerebellar and brain stem areas showed less signal. Since there was no difference in CD68 signal intensity in hypoxic patients compared to the rest of the cohort, IHC-detection methodology was considered sufficient.

We compared patients 5, 9, 13, and 15 to control patients 4 and 5, which all underwent cardio-pulmonal reanimation. Figure 4B underlines that the control patients show lower microglia/macrophage activity, emphasizing that hypoxic damage is not a confounder to the described neuropathology.

### 3.6. Inflammatory lesions are still present but less pronounced in post COVID-19 patients

**Figure 5:**
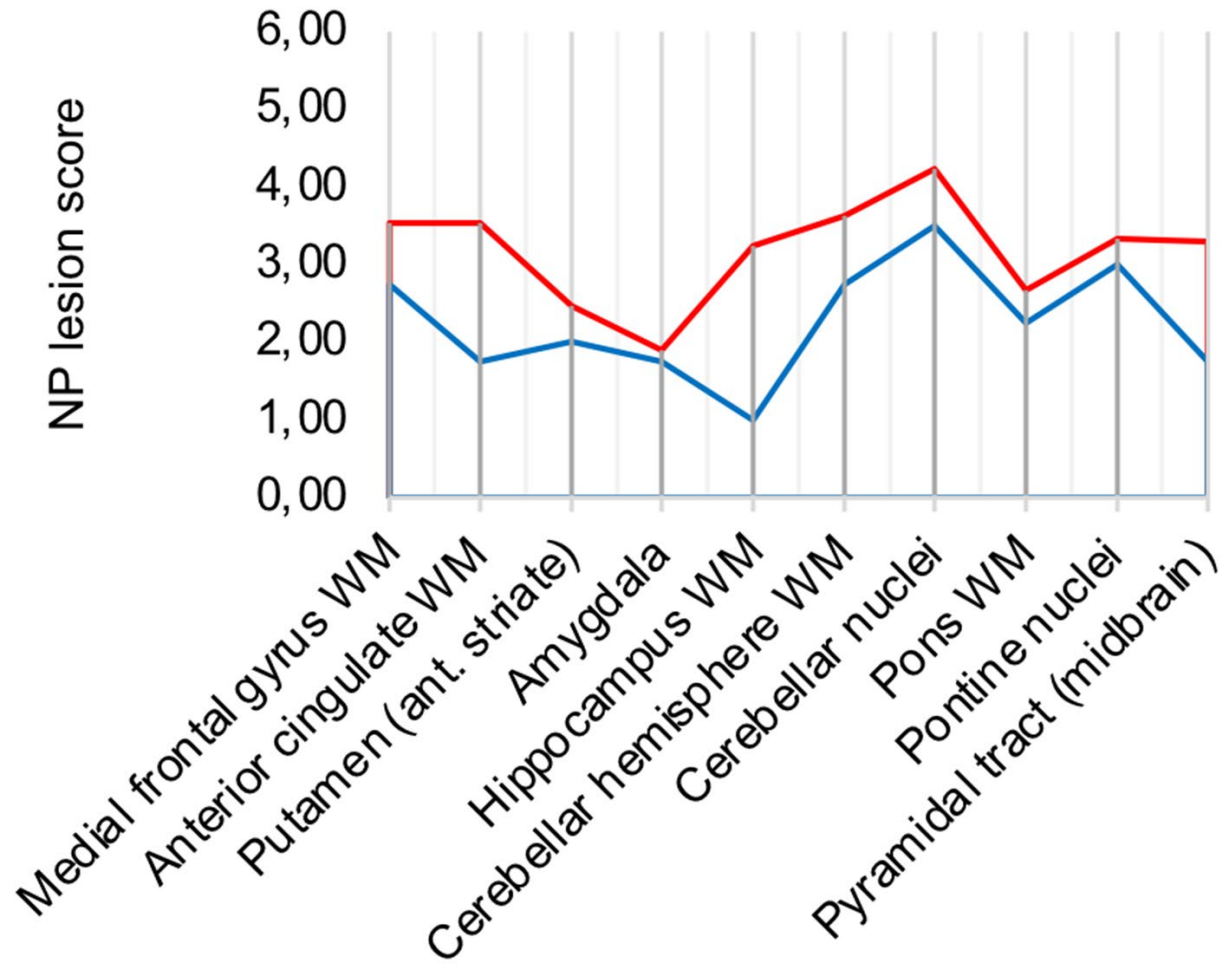
Lesion pattern of acute COVID-19 (red) vs. post COVID patients (blue).

Two out of the 17 COVID-19 group patients died unrelated to the SARS-CoV-2 infection and had reported only mild COVID-19 symptoms. Patient 6 died of pyelonephritis and had the first positive SARS-CoV-2 testing 10 weeks before. Patient 17 died of acute liver failure after surgery and had the first positive SARS-CoV-2 testing 4–5 weeks before. Both patients were compared to the remaining 15 patients of the cohort (illustrated in Figure 5). While lesions in the cerebellum and brain stem were similar to acute COVID-19, microglia/macrophage activity in the white matter of the cerebrum was less pronounced.

### 3.7. Immunohistochemistry yields no reliable detection of virus antigens

We tried to detect viral proteins using commercially available antibodies (Table 2) for immunohistochemistry and compared the results to controls.

**Figure 6:**
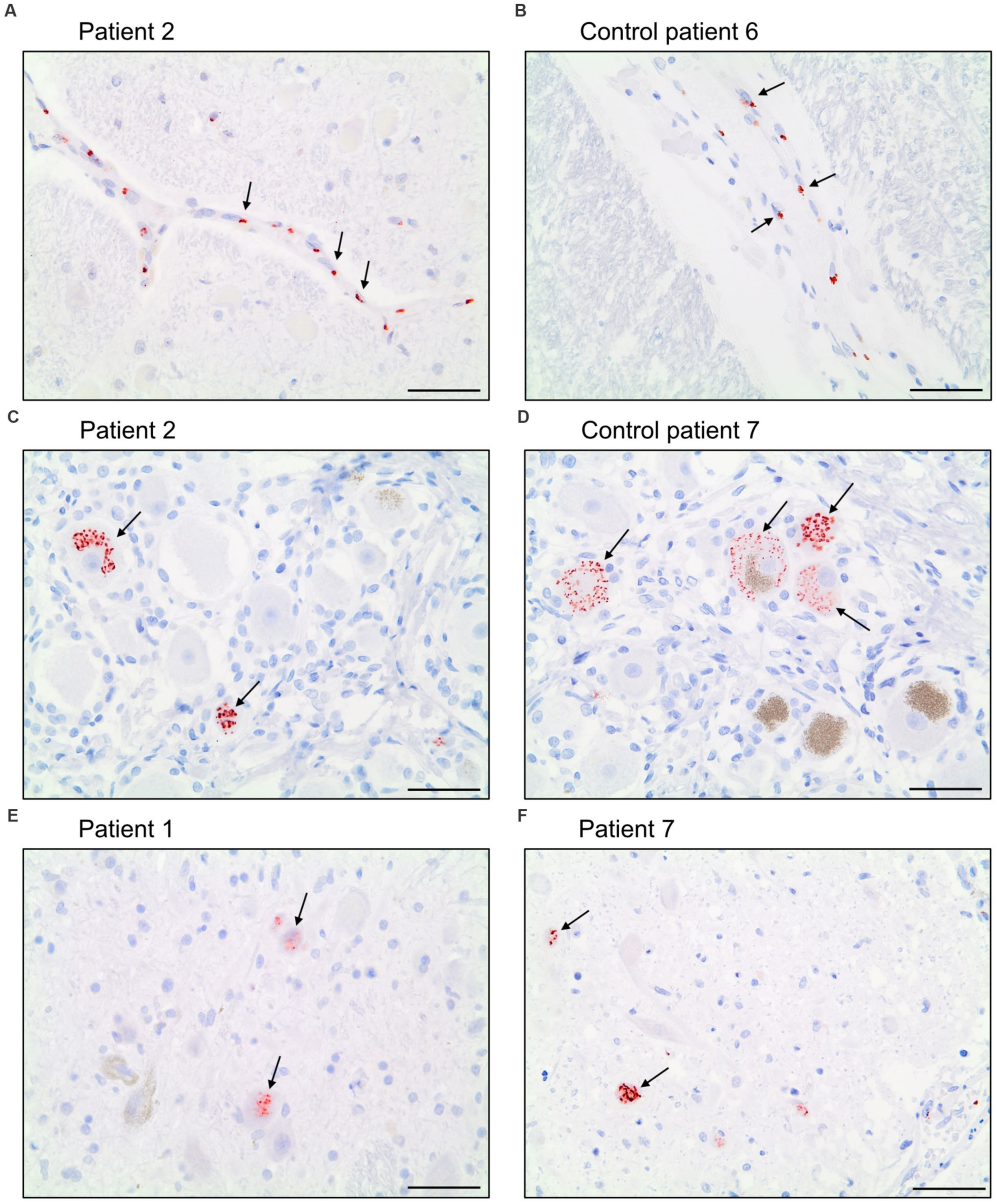
Immunohistochemical staining pattern of antibody Abcam 3A2 directed against SARS-spike glycoprotein. In the cytoplasm of cerebral endothelial cells fine, granular structures were detected in COVID-19 patients **(A)** and control patients **(B)**. In neurons of the trigeminal ganglion a granular cytoplasmic staining different from lipofuscin granula was visualized in COVID-19- **(C)** and control patients **(D)** as well as in some neurons of nuclei of the vagus nerve of COVID-19 patients **(E,F)**. Scale bars = 50 microns.

The antibody Abcam 3A2 marked fine, granular structures in the cytoplasm of cerebral endothelial cells, though only in a subset of vessel sections. In some neurons of the trigeminal ganglion, it marked cytoplasmatic granula different from lipofuscin granula. Since both pattern (illustrated in Figure 6) could also be observed in control patients who died before the onset of the pandemic, it is likely that Abcam 3A2 binds a non-SARS-CoV-2 specific antigen.

Antibody Abcam 3A2 also marked cytoplasmatic granula in some neurons of the nuclei of the vagus nerve. This pattern could not be reproduced in control patients. Antibody SB-NC yielded neither a signal in COVID-19 nor in control patients. A specific binding of antibody SB-NC to SARS-CoV-2 nucleocapsid protein could therefore not be reproduced. Antibody CoV-2-S1A9 produced no neuronal signal. However, it showed a cytoplasmatic reaction pattern in endothelial cells. This pattern was also reproduced in control patients. Antibody SB-Spike also marked cytoplasmatic structures in endothelial cells of COVID-19 patients. Antibody Novus-NC marked the cytoplasm of some endothelial cells and meningeal macrophages. Both patterns were also reproduced in control patients.

In conclusion, commercially available antibodies for detection of SARS-CoV-2 virus material in immunohistochemistry yielded no specific signal over controls.

## 4. Discussion

### 4.1. Microglia/macrophage activation is not COVID-19 specific

In this study, COVID-19 neuropathology presented as a pathology of the white matter as well as the brain stem and cerebellum. Throughout all analyzed patients, there was diffuse parenchymal microglial activity and pronounced perivascular macrophage activation in those areas. Our findings are consistent with observations of Matschke et al. (7) who described CD68-positive microglial activity in the brain stem and cerebellum with only little involvement of the frontal lobe (7). Thakur et al. (15) and Poloni et al. (16) also described activated microglia and formation of nodules in the brain stem (15, 16).

Similar to the findings of Gelpi et al. (17), COVID-19 patients of this study showed diffuse parenchymal microglial activity in the white matter at large. However, the fact that diffuse microglial activity in white matter areas of the cerebrum was especially distinct in cases with a severe course of disease as well as control case 1 with influenza, leads to believe that neuroinflammation in this area might not be COVID-19 specific but rather consequence of a cytokine storm. Gelpi et al. (17) also described similarities in microglial activation between COVID-19 and influenza. In contrast, Poloni et al. (16) attribute microglial activity in the frontal lobe of COVID-19 patients to neurodegenerative processes as a co-pathology (16). Matschke et al. (7) describe similarities in the pattern of diffuse, nodular microglia in COVID-19 to other cases of viral or autoimmune encephalitis (7).

### 4.2. Macrophage clusters – sign of microinfarction?

CD68-immunoreactive clusters were detected in 10 of 17 COVID-19 patients representing 59% of patients. Matschke et al. (7) also described similar clusters in the brain stem and surrounding parenchyma (7). In the context of COVID-19 infection, hypercoagulability and frequent arterial and venous embolisms and thrombi are well documented (18, 19). In combination with endothelitis, which has also been associated with COVID-19, clusters can likely be attributed to microinfarction (20, 21). Supporting this hypothesis, Cosentino et al. summarized neuropathological findings of 438 COVID-19 autopsies from 45 publications and saw hypoxic–ischemic lesions in 40.8% of cases (10).

However, we neither found signs of endothelitis nor frequent ischemic lesions in form of apoptotic or necrotic tissue changes in close proximity to clusters, although some axonal damage was detectable. Statistical analysis of COVID-19 patients showed that both the diffuse parenchymal microglial pattern and the perivascular macrophage pattern were elevated relative to other patients in areas with clusters. It is therefore an alternative explanation that clusters represent the end stage of microglial/macrophage activation in the white matter.

### 4.3. Neuropathology shows no correlation to severity of disease

Some COVID-19 patients in our cohort suffered a severe course of disease. We found evidence of intensive care treatment, such as septic embolisms and signs of acute perimortal hypoxia after cardiopulmonary reanimation. However, we were unable to correlate the intensity of microglia and macrophage activity to clinical severity of disease. Au contraire, both groups presented the whole spectrum of microglial activation. We found severe microglia/macrophage reaction in patients with “mild course of disease” and mild activity in those who were clinically severely affected.

Poloni et al. (16) also could not find a difference in microglial activation in the absence or presence of severe bacterial superinfection (16). We therefore assume that COVID-19-related cytokine storms lead to endothelial dysfunction and crossing of neuroinflammatory activity through the blood–brain-barrier of the central nervous system regardless of clinical burden.

### 4.4. Remarkable similarity to severe influenza

Attributing microglial activation caused by cytokine storms as the main cause of COVID-19 neuropathology also explains remarkable similarities to Influenza control patient 1, which also presented macrophage clusters. Cytokine storms have not only been discussed in COVID-19, but also in severe Influenza (22). In mice, Sadasivan et al. were even able to link H1N1-infection to persisting microgliosis and possible formation of neurodegenerative disorders (23).

### 4.5. Explanation for long-COVID symptoms?

Two analyzed subjects were “post COVID patients” according to the CDC’s definition (14) and died 4–5 and 10 weeks after first positive testing. In both typical “post COVID symptoms” such as fatigue, muscle aches, alteration of smell and taste, headaches, difficulty in concentration, daytime sleepiness and lightheadedness (24) have not been reported in the clinical records. However, both presented the already described neuropathological pattern of diffuse microglial and macrophage activation, although in a less marked intensity.

Results of our study show no correlation between intensity of microglia and macrophage activity and clinical severity of disease. Similarly, Douaud et al. investigated brain changes in patients before and about 141 days after SARS-CoV-2 infection in their longitudinal MRI study of UK Biobank participants (25) and found significant structural changes and larger cognitive decline in tests, even after excluding hospitalized cases.

Neuroanatomical localization of described lesions could explain some “post COVID symptoms”: Intense microglial-macrophage activity in nuclei and white matter of the cerebellum are likely cause of dizziness. Extensive microgliosis in the brain stem, possibly in important control centers of arousal and consciousness like the reticular formation, might explain difficulty in concentration in many reported “long COVID” patients with chronic fatigue.

### 4.6. Controversy of virus detection

Neurotropism of other human coronaviruses has been well documented (26–29). To date, however, detection of SARS-CoV-2 in the central nervous system of patients has been inconsistent at best: Matschke et al. (7) reported both viral proteins and RNA in the brain (7). Meinhardt et al. (3) reported SARS-CoV-2 RNA in the olfactory bulb (3). Lebrun et al. reported SARS-CoV-2 RNA positivity in the brain by PCR in all (*n* = 18) analyzed cases, while only one patient harbored SARS-CoV-2 viral proteins in the brain tissue (30). Solomon et al. (11) achieved mixed results and argued that samples might have been contaminated by blood (11). A literature review of Cosentino et al. came to the same conclusion (10). Poloni, Moretti et al. (31) detected viral antigens predominantly in lung and kidney samples where SARS-CoV-2 replicates, while brain and heart tissues presented less viral antigens. The authors conclude that SARS-CoV-2 is cleared from tissues quickly after acute infection (31).

Yang et al. were also unable to detect SARS-CoV-2 specific RNA using specimens of our collective (8). However, this does not mean that described neuropathology is necessarily unspecific: Virus detection in cases of herpes-, arbo- or enterovirus encephalitis is rarely successful, as Krey et al. (29) point out.

In light of controversy regarding virus detection, it is astonishing that specificity of commercially available antibodies for IHC-detection of SARS-CoV-2 has not been reviewed more critically. While some publications compare IHC detection to *in-situ*-hybridization, usually lung tissue was used and samples from the brain are missing (32, 33).

According to the manufacturer, antibody Abcam 3A2 binds the SARS spike glycoprotein. Meinhardt et al. (3) used the antibody to detect SARS-CoV-2 spike protein in the olfactory mucosa (3). Schwabenland et al. showed marked endothelial cells using this antibody (34). Results of our study show that antibody Abcam 3A2 likely binds to a non-SARS-CoV-2-specific antigen.

Antibody SB-NC binds the SARS-CoV nucleocapsid protein and shows cross-reactivity with SARS-CoV-2 nucleocapsid protein, according to the data sheet. It is validated for use in immunohistochemistry, though cross-reactivity has only been observed in ELISA- and Western Blot detection methods, according to the manufacturer. However, Berezowska et al. (35) and Liu et al. (36) showed successful detection of infected cells in immunohistochemistry. In our study, the antibody yielded no signal in COVID-19 and control patients. A specific binding of antibody SB-NC to SARS-CoV-2 nucleocapsid protein could therefore not be reproduced.

Antibody CoV-2-S1A9 detects the spike protein of both SARS-CoV and SARS-CoV-2 in immunohistochemistry, according to the manufacturer’s data sheet. Song et al. showed marked neurons and endothelial cells using CoV-2-S1A9 (37). Matschke et al. (7) also published marked neurons using this antibody (7). In our study, CoV-2-S1A9 produced no neuronal signal. However, it showed a cytoplasmatic reaction pattern in endothelial cells. This pattern was also reproduced in control patients.

In light of these unconclusive results, future studies should consider the limited specificity of these antibodies and add other detection methods.

Antibodies used in our study mostly showed endothelial pattern. It is likely that marked epitopes are structurally similar to SARS-CoV-2 antigens. Since SARS-CoV-2 negative controls also show these pattern, it might be that antibodies cross-react with different endemic human coronaviruses (HCoVs), which seem to have neurotropic and neuroinvasive capabilities (38). Another explanation is that antibodies cross-react with lysosome-related structures, which needs to be investigated further in the future.

### 4.7. Methodology discussion and limitations

In the present study we analyzed specimen from 17 COVID-19 patients. Comparing our cohort to sample sizes in systematic reviews from Maiese et al. (39) (21 papers, 9 cases per paper on average) and Cosentino et al. (10) (45 papers, 10 cases per paper on average), it was sufficiently large to reproduce a specific lesion pattern.

We chose immunohistochemistry to both detect protein structures and gain information on their anatomical localization, compared to the sole detection of RNA molecules by PCR.

When using postmortem specimen for immunohistochemistry, influencing factors such as autolysis and insufficient formalin fixation must be considered. It is possible that some epitopes were masked and therefore not detected by antibodies (40, 41). Epitope denaturalization through processes of antigen retrieval and insufficient blocking of endogenous peroxidases should also be considered as sources of error. However, results were persistent through multiple different pretreatment protocols to enhance IHC-detection, such as casein pretreatment and prolonged blocking of peroxidases. The fact that tissues from COVID-19 patients and control patients showed the same reaction pattern makes errors in methodology less likely.

Due to limited clinical data, correlation of neuropathological findings to patient history, especially development of neurological symptoms, was only partially possible. However, quality and quantity of data is comparable to other studies (39). Due to low case numbers, analysis was limited to the descriptive level and semiquantitative scoring, comparable to the methodology of Matschke et al. (7), Meinhardt et al. (3) and other neuropathological studies.

Since specimen collection ended in June 2021, results of this study can only be applied to the original SARS-CoV-2 variant. Neuropathological lesions in patients infected with the omicron-variant of SARS-CoV-2 might differ.

As in all autopsy-based studies, described pathology refers to the most severe cases (10). Our cohort was mostly elderly patients with multiple chronic comorbidities (age of 74.5 years on average). However, we also analyzed cases of younger patients that unexpectedly developed a severe course of disease. In light of these circumstances, the results of this study are of weak extern validity and can only be cautiously applied to younger, asymptomatic patients.

## 5. Conclusion and outlook

COVID-19 is not just a respiratory infection. Neurological symptoms of acute infection and “post-COVID” can hypothetically be explained by neuropathologic findings. We presented enhanced microglial and macrophage activity in the white matter, cerebellum and brain stem throughout patients, regardless of disease severity or neurological clinic. Microglial and macrophage activation also remained elevated in “post-COVID” patients. Like most published papers on the subject, we could not verify the presence of SARS-CoV-2-specific antigens in the brain.

Results of this study highlight the necessity to continue research on “long COVID” symptoms and pathophysiology, as SARS-CoV-2 variants become endemic and seropositivity increases.

## Data availability statement

The raw data supporting the conclusions of this article will be made available by the authors, without undue reservation.

## Ethics statement

The studies involving human participants were reviewed and approved by Saarland Chamber of Physicians. The patients/participants provided their written informed consent to participate in this study.

## Author contributions

WS-S, JS, and MK provided and organized tissue samples. WS-S administrated and supervised the project, provided the resources, captured images, reviewed, and edited the original draft. JS performed immunohistochemical and HE stains, wrote the original draft, and created visualizations. MK performed immunohistochemical stains. WS-S and JS performed microscopic analysis. SS provided data, reviewed, and edited the manuscript. All authors have read and approved the manuscript.

## Conflict of interest

The authors declare that the research was conducted in the absence of any commercial or financial relationships that could be construed as a potential conflict of interest.

## Publisher’s note

All claims expressed in this article are solely those of the authors and do not necessarily represent those of their affiliated organizations, or those of the publisher, the editors and the reviewers. Any product that may be evaluated in this article, or claim that may be made by its manufacturer, is not guaranteed or endorsed by the publisher.
